# Engineering topological interface states in metal-wire waveguides for broadband terahertz signal processing

**DOI:** 10.1515/nanoph-2023-0900

**Published:** 2024-04-15

**Authors:** Mohammad Ghazialsharif, Junliang Dong, Domenico Bongiovanni, Anton Vorobiov, Ziteng Wang, Zhigang Chen, Detlef Kip, Roberto Morandotti

**Affiliations:** 14851 Institut national de la recherche scientifique, Centre Énergie Matériaux Télécommunications, Varennes, QC J3X 1P7, Canada; 12538 The MOE Key Laboratory of Weak-Light Nonlinear Photonics, TEDA Applied Physics Institute and School of Physics, Nankai University, Tianjin 300457, China; Faculty of Electrical Engineering, 26554 Helmut Schmidt University, Holstenhofweg 85, 22043 Hamburg, Germany

**Keywords:** terahertz, topological interface states, Zak phase, waveguides, analog signal processing

## Abstract

Innovative terahertz waveguides are in high demand to serve as a versatile platform for transporting and manipulating terahertz signals for the full deployment of future six-generation (6G) communication systems. Metal-wire waveguides have emerged as promising candidates, offering the crucial advantage of sustaining low-loss and low-dispersion propagation of broadband terahertz pulses. Recent advances have opened up new avenues for implementing signal-processing functionalities within metal-wire waveguides by directly engraving grooves along the wire surfaces. However, the challenge remains to design novel groove structures to unlock unprecedented signal-processing functionalities. In this study, we report a plasmonic signal processor by engineering topological interface states within a terahertz two-wire waveguide. We construct the interface by connecting two multiscale groove structures with distinct topological invariants, i.e., featuring a π-shift difference in the Zak phases. The existence of this topological interface within the waveguide is experimentally validated by investigating the transmission spectrum, revealing a prominent transmission peak in the center of the topological bandgap. Remarkably, we show that this resonance is highly robust against structural disorders, and its quality factor can be flexibly controlled. This unique feature not only facilitates essential functions such as band filtering and isolating but also promises to serve as a linear differential equation solver. Our approach paves the way for the development of new-generation all-optical analog signal processors tailored for future terahertz networks, featuring remarkable structural simplicity, ultrafast processing speeds, as well as highly reliable performance.

## 1 Introduction

Terahertz (THz) radiation, spanning the electromagnetic spectrum from 0.1 THz to 10 THz, represents a transformative technology poised to become the cornerstone of future 6G networks [1], [2]. Unlike millimeter waves used in the 5G networks, THz waves introduce a significant leap in bandwidth, capable of addressing the ever-increasing demands for higher data rates, targeting terabits-per-second (Tb/s). This remarkable bandwidth expansion necessitates the development of a new generation of physical-layer components [3], including switches, couplers, filters, and multiplexers, which serve as the fundamental building blocks to manipulate THz communication signals.

In recent years, various types of THz waveguides have been explored toward implementing signal-processing functionalities [4]. Among them, THz metallic waveguides that support transverse electromagnetic (TEM) modes, such as parallel-plate waveguides (PPWGs) [5], [6], [7] and metal-wire waveguides [8], [9], have garnered significant attention as viable alternatives due to their ability to facilitate low-loss and low-dispersion propagation of broadband THz pulses. Compared to the large footprint of PPWGs, THz metal-wire waveguides possess distinct advantages, such as structural simplicity [10], tolerance to bending [11], and compatibility with cables for efficient and straightforward connections [12]. However, while metal-wire waveguides excel in guiding THz signals, unlocking their potential for signal-processing functionalities remains a challenge. This is because the tight confinement of modal energy within the wavelength-scale space between the wires significantly limits the available methods for manipulating propagating THz waves. To this end, our group proposed a universe approach for the realization of broadband THz signal processing in metal-wire waveguides by directly engineering the wire surfaces [13]. In principle, the THz guidance in metal-wire waveguides fundamentally relies on the propagation of surface plasmon polaritons (SPPs) along the metal–air interface [14], which is extremely sensitive to the metal surface conditions. When a periodic array of grooves is engraved on the metal surface, surface states behaving like SPPs, so-called spoof SPPs, can still be supported [15]. The geometry of these grooves can be judiciously designed to tailor the THz propagation characteristics. As an example, we demonstrated that by etching grooves with so-called “multiscale structures” onto the wires within a two-wire waveguide (TWWG), Bragg gratings can be achieved without the need to introduce additional materials [13]. Such an approach opens up new perspectives for the realization of waveguide-integrated signal processors with remarkable structural simplicity, achieved by engineering innovative structures directly on the wires.

The concept of multiscale structures, obtained by introducing a wavelength-scale periodic modulation onto subwavelength-scale periodic grooves, is analogous to a photonic crystal (PC) (see Supplementary Note 1). Therefore, the location of the Bragg bandgap that appears in the TWWG with multiscale-structured grooves can be tuned by varying the period of the wavelength-scale modulation, corresponding to the geometry of the unit cell in a PC. As well, such a concept paves the way to introduce topological interface states into metal-wire waveguides for broadband THz signal processing. In principle, a topological interface state can be formed between two PCs with different topological properties [16], [17]. This intriguing occurrence is pronounced within the context of a 1D lattice structure, rooted in the classic Su–Schrieffer–Heeger (SSH) model [18]. Nowadays, due to their robust transport of light and immunity to disorders or irregularities, topological interface states in photonic structures [19], [20], [21], [22] have found a plethora of applications, spanning from cavities [23], filters [24], splitters [25], and switches [26]. Furthermore, topological interface states allow flexible control of the quality factor (Q-factor) in resonances, thus promising a diverse array of signal-processing functionalities.

In this paper, we report a THz plasmonic signal processor by engineering topological interface states within a TWWG. We construct an SSH-like model using the concept of multiscale structures and introduce an interface by connecting two finite multiscale structures with distinct topological properties. We confirm the existence of the topological interface state by numerical simulations and experimental investigation of the transmission spectrum. In particular, the achieved transmission spectrum features a transmission peak within the forbidden band, with the Q-factor being controllable by judiciously adjusting the geometry of the grooves and the number of unit cells. In addition, we demonstrate that the resonance features can be further tailored by cascading multiple topological interface states within the TWWG. Our results demonstrate the potential of implementing topological interface states in developing new-generation ultrafast and highly reliable THz plasmonic analog signal processors.

## 2 Theoretical background

**Figure 1:**
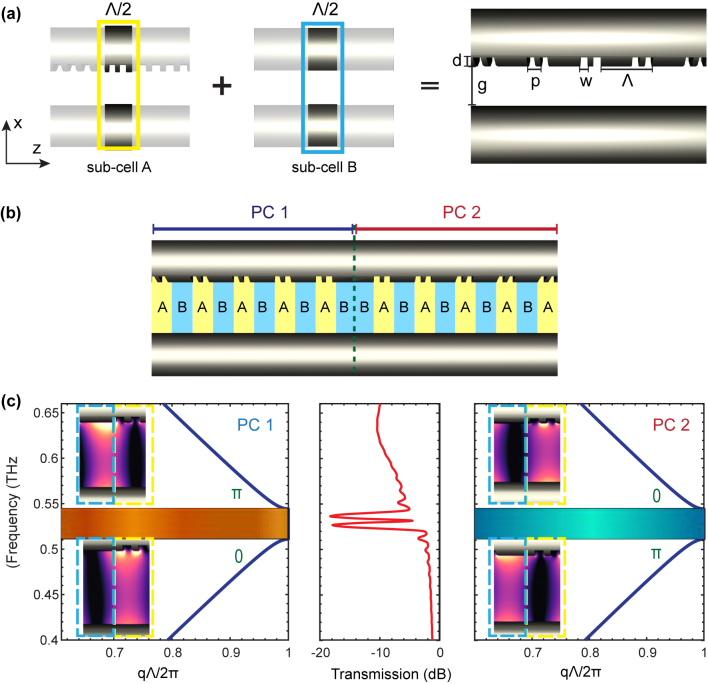
Constructing the topological interface state within a TWWG based on multiscale structures. (a) Schematic of the 3D TWWG model with uniform multiscale-structured grooves. This structure is achieved by superimposing a wavelength-scale periodic modulation onto the sub-wavelength-scale periodic grooves, which can be interpreted as the combination of subcells (A and B) with different propagation constants. (b) Schematic of connecting two finite PCs with different initial subcells: PC1 starts with subcell A (unit cell “AB”) and PC2 starts with subcell B (unit cell “BA”). (c) Band structures for PC1 and PC2, along with the transmission spectrum of the two connected multiscale structures. The Zak phase is marked for each band in green. The THz electric field modulus profiles at the band-edges (0.525 THz and 0.535 THz) shown in the insets demonstrate different parities due to the difference in Zak phases.

A multiscale structure within a TWWG (with a wire radius *r* of 127 µm and an air gap *g* of 300 µm) is achieved by superimposing a wavelength-scale periodic modulation Λ onto the subwavelength-scale periodic grooves directly engraved along one of the two wires. As shown in Figure 1(a), such a multiscale structure can be interpreted as the combination of two subcells with different propagation constants *k*_*i*_, where the subscripts *i* = *A*, *B* correspond to each individual subcell. Any geometrical difference (depending on the subwavelength-scale groove width *w*, depth *d*, and period *p*) between the subcells results in variations in the effective refractive index *n*_*i*_, leading to a periodic modulation at the wavelength scale Λ. Analogous to 1D PC lattices, the bandgap structure of such a multiscale structure can be obtained from the Bloch theorem [27]:

(1) $$\begin{array}{ll} \cos\left(q\Lambda \right) & =\cos k_{A}d_{A}\cos k_{B}d_{B}\\ & \;-\frac{1}{2}\left(\frac{n_{B}}{n_{A}}+\frac{n_{A}}{n_{B}}\right)\sin k_{A}d_{A}\sin k_{B}d_{B} \end{array}$$

The propagation constant is *k*_*i*_ = *ωn*_*i*_/*c*, where *c* is the speed of light, and *q* is the Bloch wave vector. Considering our design in Figure 1(a), we have *d* = 40 µm, *w* = 40 µm, *p* = 80 µm, and Λ = 280 µm. Based on Eq. (1), the achieved band structure within the TWWG is shown in Figure 1(c). The bandgap occurs at *f*_bandgap_ = *c*/2Λ = 0.53 THz, which satisfies the Bragg condition. By simply altering the period of the wavelength-scale modulation Λ, the location of such a Bragg bandgap can be tuned within a bandwidth as large as ∼1 THz.

It is noteworthy that Eq. (1) demonstrates the bandgap structures of an infinite PC system. However, according to the PC bandgap theory, a finite PC system can potentially exhibit distinct topological invariant properties in contrast to an infinite similar PC system [28]. Zak phases, which are considered the most important parameter to describe the topological properties of the band structure for 1D PCs, are defined as [29], [30]:

(2) $$\theta_{m}^{\text{Zak}}=\int_{-\pi /\Lambda }^{\pi /\Lambda }\left[i\int_{\text{unit cell}}dz\varepsilon \left(z\right)u_{m,q}^{*}\left(z\right)\partial_{q}u_{m,q}\left(z\right)\right]dq,$$

where $u_{m,q}\left(z\right)$ is the periodic-in-cell part of the Bloch electric field eigenfunction of the state on the *m* ^th^ band with the wave vector *q*, and *ε*(*z*) is the relative permittivity. Based on Eq. (2), it is obvious that the value of $\theta_{m}^{\text{Zak}}$ depends on the initial point of its integral period, i.e., the origin of the unit cell in a finite PC system. Considering our multiscale structure, the difference in $\theta_{m}^{\text{Zak}}$ is π, when the origin of the unit cell is chosen to be either subcell A (unit cell “AB”) or subcell B (unit cell “BA”) [17]. As shown in Figure 1(b), two similar finite PC systems with different origins of the unit cell exhibit a common photonic band structure but are distinct in terms of the topological invariant, i.e., a difference of π in the Zak phases of bulk bands. The corresponding Zak phase of each band is highlighted in green. Furthermore, by following a mirror symmetry and connecting two finite PCs with π-shift Zak phases, a topological interface can be constructed. While a conventional PC blocks the energy transmission within its band gap, a PC with an interface state allows energy propagation through the band gap. Such an occurrence can be explained by investigating the surface impedances *Z*_*s*_ of each PC system. In principle, *Z*_*s*_ is a pure imaginary number inside a band gap, i.e., $Z_{s}^{\left(m\right)}=i\xi^{\left(m\right)}$ , where $\xi^{\left(m\right)}$ is a real number, which shows the impedance value of the *m* ^th^ gap [31]. In particular, the sign of $\xi^{\left(m\right)}$ relies on the sum of Zak phases of all the isolated bands. Accordingly, when our multiscale structure starts with subcell A, $\xi^{\left(m\right)}<0$ ; when our multiscale structure starts with subcell B, $\xi^{\left(m\right)}>0$ . Therefore, by connecting two multiscale structures with unit cells starting with different subcells, we construct an interface in the bandgap with equal surface impedance values but opposite signs, i.e., *Z*_*s*1_ + *Z*_*s*2_ = 0, in turn leading to energy transmission at the interface [32].

## 3 Results and discussions

In our study, we connect two multiscale structures starting with different subcells within a TWWG. As depicted in Figure 1(b), the right structure (PC2, unit cell “BA”) is mirror-symmetric to the left one (PC1, unit cell “AB”), which consists of the inverted unit cells. Since the wavelength-scale periodic modulations Λ of the left and right structures are the same, the locations of the bandgap are not altered during the inversion process. However, the two multiscale structures possess distinct topological invariants, i.e., a π-shift difference in Zak phases, in turn resulting in a topological interface when connecting the two of them (see Supplementary Note 2).

**Figure 2:**
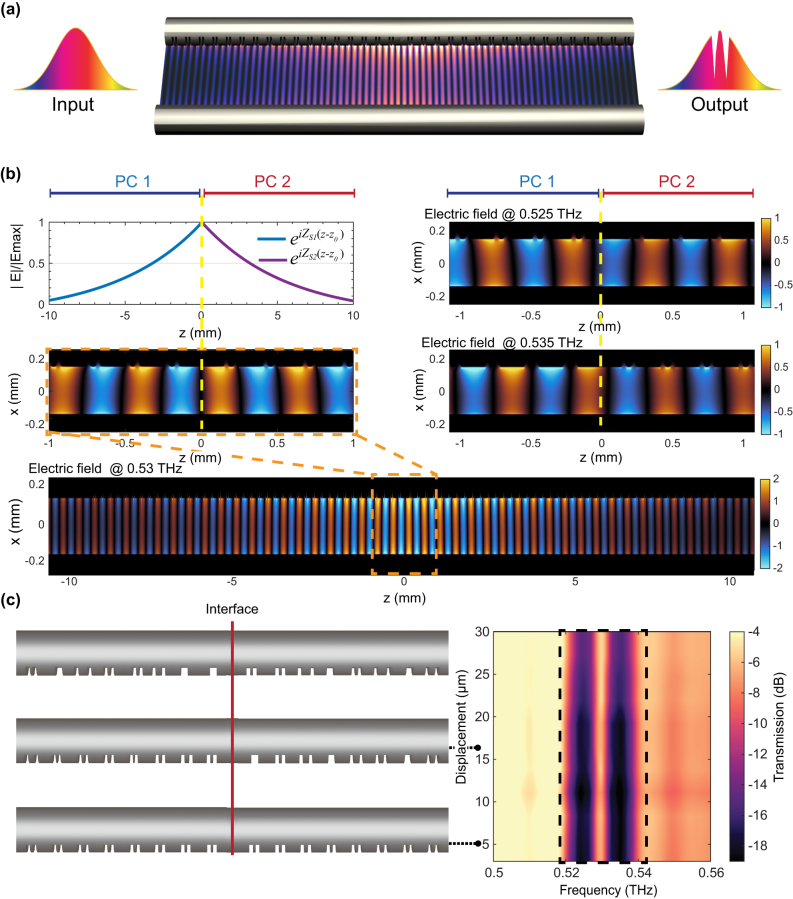
Numerical investigation of the topological interface state within a TWWG. (a) Schematic of the THz electric field modulus distribution of the topological interface state in the TWWG. (b) The zoom-in section of the THz electric field distributions at the topological interface (at 0.53 THz) and at the band edges (at 0.525 THz and 0.535 THz). The electric field profile at 0.53 THz illustrates the symmetry of the energy distribution at the topological interface, while the profiles at 0.525 THz and 0.535 THz exhibit nonsymmetrical distributions due to the phase transition at the topological interface. (c) The achieved transmission spectrum when random disorder is introduced. In both cases, the transmission spectra demonstrate the robustness of the locations of the transmission peak (averaged over 10 realizations of disorder) while randomly shifting the groove positions.

We first numerically investigate the proposed structure (with identical geometric parameters defined in the previous section) via finite-difference-time-domain (FDTD) simulations. The number of unit cells on both sides is set to be 40, leading to a total length of 80Λ. The simulated transmission spectrum, as illustrated in Figure 1(c), features a transmission peak in the center of the forbidden band at 0.53 THz and thus, confirms the existence of the topological interface state. Note that additional losses are observed in the higher frequency region (∼0.6 THz) of the transmission spectrum. This is because the grooves were engraved along one side of the two wires; consequently, the phase shift between the THz electric fields propagating along the nonsymmetric structures leads to additional losses [13]. The THz electric field modulus distribution of the topological interface state is shown in Figure 2(a), which is localized near the vicinity of the interface. In addition, it is obvious that the THz electric field experiences an exponential increase on the left side of the interface and decays exponentially on the right side of the interface [33], [34]. This is because the propagating THz field *E* along the *z*-direction within the TWWG can be expressed as $\vec{E}=\left|E\right|{\text{e}}^{\text{i}Z_{s}\left(z-z_{0}\right)}$ , where *z*_0_ is the PC starting point; therefore, the electric field increases exponentially when *ξ* < 0 and decays exponentially when *ξ* > 0, corresponding to the π-shift in the Zak phase at the interface. Furthermore, the topological phase transition can be confirmed by the changes in the symmetries of the interface states. As shown in Figure 2(b), unlike the symmetric energy distribution observed at the interface state (0.53 THz), the THz electric field distribution at the lower (0.525 THz) and upper (0.535 THz) band edges exhibit a nonsymmetrical energy distribution, indicating the occurrence of a phase transition at the topological interface. In addition, the amplitude of the electric field shows different parities around the interface: (1) the left multiscale structure (PC 1) has the maximum magnitude in subcell A at the lower band edge and a zero magnitude in subcell A at the upper band edge; (2) the right multiscale structure (PC2) has a zero magnitude in subcell A at the lower band edge and the maximum magnitude in subcell A at the upper band edge, as highlighted in the insets of Figure 1(c). The existence of the topological interface states can also be predicted when the states at the lower band edge for the common bandgap of the left and right structures belong to different types.

One of the most prominent characteristics of topological interface states is their inherent protection against disorders or irregularities [35]. The PC lattice in Figure 1(b) has two mirror centers, i.e., the centers of the subcells A and B, where the edge cutting is conducted at the start of subcell A (B) for PC1 (PC2). The mirror symmetry contributes to the topological protection of interface states. To validate this property, we deliberately introduce disorders into the structure by randomly shifting the groove positions along the wire surface. We limit the groove displacements within ±30 µm because, in some instances, the adjacent grooves would be combined, as shown in Figure 2(c). Disorders with the same strength are randomly introduced into 10 samples, and we subsequently examine the averaged transmission spectra to assess the topological protection against the random variations of the groove positions. Our simulation results shown in Figure 2(c) suggest topological protection, since the locations of the transmission peak at the center of the forbidden band barely change as the disorder strength increases. Under such protection, the requirement for manufacturing precision could be reduced, leading to the realization of samples with immunity to fabrication imperfections.

**Figure 3:**
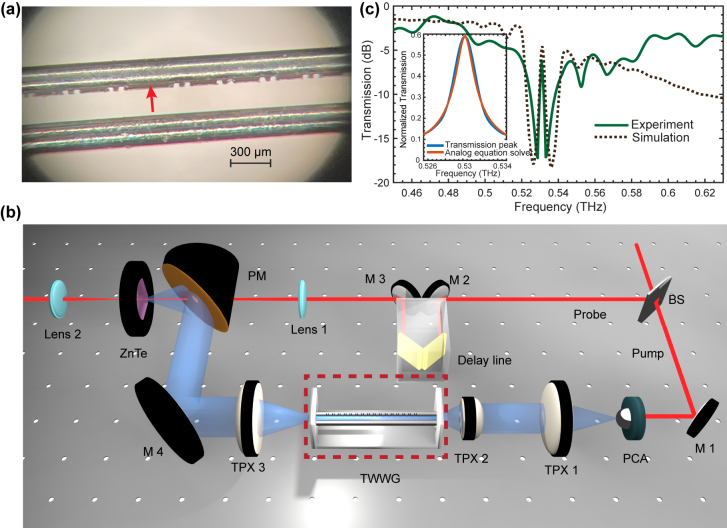
Experimental characterization of the topological interface state within the TWWG. (a) Optical microscopic image of the fabricated TWWG sample, focusing on the section of the topological interface. (b) Schematic of the experimental THz-TDS setup. PCA, photoconductive antenna; TPX, THz TPX lens; BS, beam splitter; M, mirror; and PM, parabolic mirror. (c) Comparison between the simulated and experimental transmission spectra of the TWWG. The occurrence of the transmission peak within the forbidden band at 0.53 THz confirms the existence of the topological interface state. The inset demonstrates how the achieved resonance fits the transfer function of a first-order differential equation.

We fabricate the designed samples by engraving grooves on bare stainless-steel wires using diamond blades. First, the wires are attached to a flat silicon wafer into which a small V-groove has been cut to keep the wires straight and flat. Then the silicon wafer with wires is installed on the base of an automatic dicing saw. The thickness of the employed diamond blade was 40 µm, which determines the groove width. The grooves along the wires are engraved by utilizing a three-dimensional motor control of the dicing saw. Deviations in the depth are mainly induced by the blade wearing over several cuts. The width of the grooves is constant except for a few burrs at the edge of the cuts. To mount the sample, two polymer slabs with holes are used to hold and support them. The diameter of the holes is set to 813 µm in order to guarantee that the gap size between the wires is 300 µm. The optical microscopic image of the fabricated sample, zooming in on the section of the topological interface, is shown in Figure 3(a). We perform the experimental characterization of the sample using a customized THz time-domain spectroscopy (TDS) system [13] as illustrated in Figure 3(b). THz pulses are generated by exciting a photoconductive antenna (PCA) and then focused into the TWWG using TPX lenses. The detection of the output THz signals is achieved by implementing the electro-optic sampling technique via a ZnTe crystal with a thickness of 3 mm. Each recorded THz waveform is centered in a time window of 50 ps with a time resolution of 0.05 ps.

The experimental transmission spectrum is achieved by calculating the ratio between the power spectra of the signals passing through the TWWG with and without the designed grooves. As shown in Figure 3(c), a transmission peak is observed in the center of the forbidden band at 0.53 THz, which experimentally confirms the existence of the topological interface state within the TWWG. The minimum transmission power within the forbidden band is −18 dB and the transmission at the interface state is −4 dB, resulting in a modulation depth of −14 dB and a Q-factor of 148.1. The additional loss at the transmission peak could be attributed to the burrs at the edge of the cuts induced during the fabrication. Note that the mismatch between the simulated and experimental transmission spectra is mainly due the fabrication limitations and the uneven tightening of the wires.

Such a prominent transmission peak produced by the topological interface state is desired for analog signal processing. By setting the signal carrier frequency to the transmission peak value, various signal processing functionalities can be realized. For example, according to coupled-mode theory, the transfer function $H\left(f\right)$ of such a resonance close to its central frequency *f*_0_ can be approximated using:

(3) $$H\left(f\right)=\frac{A}{i\left(f-f_{0}\right)+f_{0}/2Q},$$

where *A* is an arbitrary constant. Notably, this function serves as the solution of a first-order linear differential equation [36]: given an input signal $g\left(t\right)$ modulated at the carrier frequency *f*_0_, i.e., $\tilde{g}\left(t\right)=g\left(t\right)\cos\left(2\pi f_{0}t\right)$ , the output of the system would be $\tilde{f}\left(t\right)=f\left(t\right)\cos\left(2\pi f_{0}t\right)$ , where $f\left(t\right)$ is the solution of the differential equation $f^{\prime }\left(t\right)+\alpha f\left(t\right)=\beta g\left(t\right)$ , with *α* = *πf*_0_/*Q* and *β* = 2*πA*. As shown in the inset of Figure 3(c), by fitting the transfer function $H\left(f\right)$ to the achieved transmission peak, we have *A* = 0.002*f*_0_ and *Q* = 148, corresponding to the parameters of the differential equation *α* = 0.007*πf*_0_ and *β* = 0.004*πf*_0_. This analysis demonstrates the possibility to realize various first-order differential equation solvers by engineering topological interface states within the TWWG. Indeed, a large range of values of the parameter *α* and *β* can be achieved by varying the geometry of the devices, by engraving distinct grooves in subcell B, as well as by adding additional loss in the waveguide.

**Figure 4:**
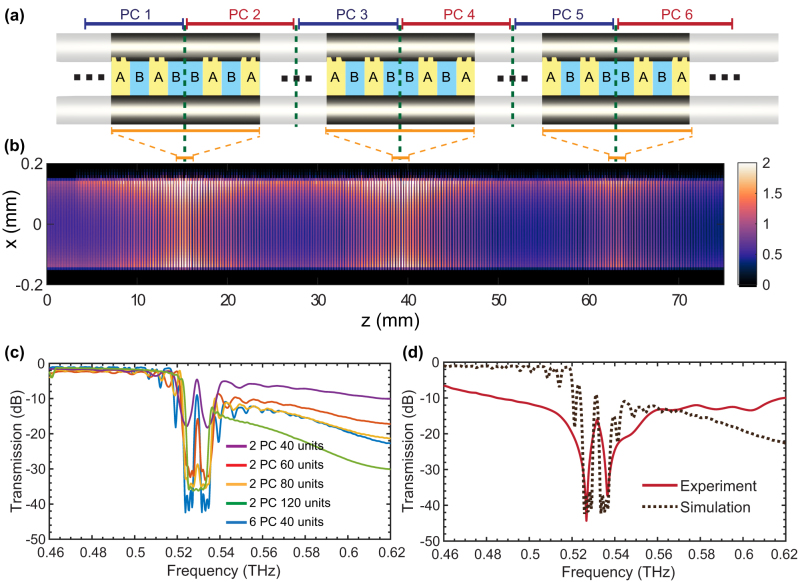
Tuning the Q-factor by cascading three topological interface states with the TWWG. (a) TWWG with three cascaded topological interface states. (b) Simulated THz electric field modulus distribution within the TWWG with three cascaded topological interface states. (c) Simulated transmission spectra with different numbers of unit cells. (d) Comparison between the simulated and experimental transmission spectra of the TWWG with three cascaded topological interface states.

Finally, we demonstrate that the modulation depth of the resonances can be enhanced by increasing the number of unit cells. Figure 4(c) provides the simulated transmission spectra at different numbers of unit cells. The transmission peak gets lower when the number of unit cells increases; when two multiscale structures with a length of 120Λ (a total length of 240Λ) are connected, no transmission peak at the interface state is observed. This is because, as the number of unit cells increases, most of the energy would reflect from the left part of the structure before reaching the topological phase transition point, leading to a reduced transmission peak. However, when cascading three topological interface states (shown in Figure 4(a)), it is possible to achieve a significant increase in transmission within the forbidden band, as shown in Figure 4(c) in blue. We constructed a lattice with a higher modulation depth by cascading six PCs (40Λ). Such a design involves cascading similar structures, each featuring an identical bandgap but with periodic inverted Zak phases. Essentially, this periodic change in Zak phases triggers the emergence of interface states at each connecting point between PCs. In addition, from the viewpoint of surface impedances, the interface state can be created by stacking several structures with periodic changes in the sign of the imaginary component of the surface impedances, satisfying the condition that *Z*_total_ = *Z*_*S*1_ + *Z*_*S*2_ + *Z*_*S*3_+ *Z*_*S*4_+ *Z*_*S*5_ + *Z*_*S*6_ = 0. The simulated THz electric field distribution in such a cascaded system, as shown in Figure 4(b), confirms the existence of the interface states at each phase transition point. We fabricate the sample based on this cascading design and perform the experimental characterization with our THz-TDS system. The achieved experimental transmission spectrum, as shown in Figure 4(d), exhibits good agreement with the simulated transmission spectrum, featuring a modulation depth of 31 dB and an enhanced Q-factor of 260. It is important to note that the concept of cascading multiple topological interface states (featuring distinct parameters) within our waveguide may enable the realization of higher-order differential equation solvers. Our results show that constructing the topological interface states based on multiscale structures offers more degrees of freedom to tailor the Q-factor and promises to realize various unprecedented signal-processing functionalities.

## 4 Conclusions

In summary, we have demonstrated the realization of topological interface states within a TWWG by directly engraving grooves along the metal-wire surfaces. By connecting two multiscale-structured grooves with distinct Zak phases, a topological interface state is constructed, leading to the occurrence of a transmission peak in the center of the topological bandgap. We have analytically discussed the origin of this topological interface state and confirmed its existence by performing both numerical simulations and experimental characterization. In addition, we have shown that the topological interface state is resistant to disorders and offers flexible control over the Q-factor. Based on these promising characteristics, engineering the topological interface states into TWWGs potentially facilitates the realization of important signal processing functionalities, such as band filtering and isolating, with remarkable structural simplicity. We have also shown the possibility of realizing linear differential equation solvers by employing topological interface states. Our approach opens up new perspectives for developing robust all-optical plasmonic analog signal processors tailored for future 6G networks, which can not only execute ultrafast high-throughput and power-efficient signal processing tasks but also effectively compete with their digital counterparts in terms of reliability and flexibility.

## Supplementary Material

Supplementary Material Details
