# Supplementary material for: Engineering topological interface states in metal-wire waveguides for broadband terahertz signal processing
Source: Nanophotonics. 2024 Apr 15;13(10):1929–37. doi: 10.1515/nanoph-2023-0900 (PMC11052534; doi:10.1515/nanoph-2023-0900)
Supplement: Supplementary file 1 — Supplementary Material Details [file j_nanoph-2023-0900_suppl_001.pdf]

Supplementary Information

Engineering topological interface states in metal-wire waveguides for broadband terahertz signal processing

Supplementary Note 1: THz metal-wire waveguides with multiscale-structured grooves

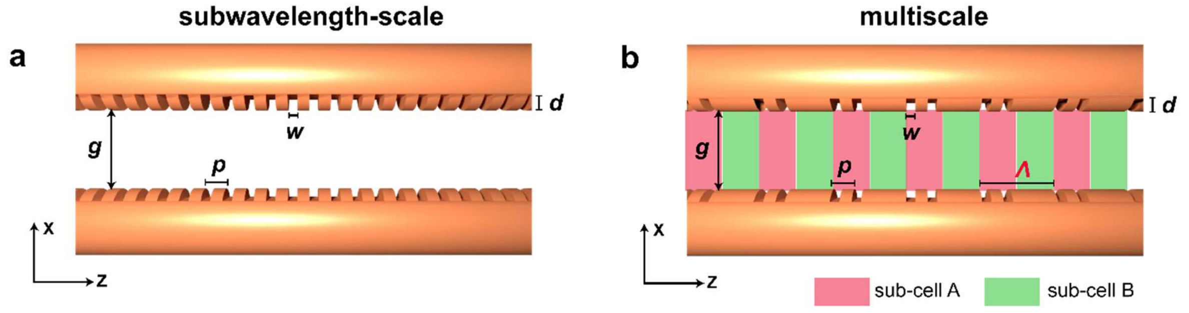

Fig. S1 (a) Schematics of the TWWG with subwavelength-scale periodic grooves. The grooves are engraved on both wires and face each other. (b) Schematics of the TWWG with multiscale grooves.

In the THz frequency regime, metals are generally considered perfect conductors, as the negligible penetration of the electromagnetic field leads to highly delocalized surface plasmon polaritons (SPPs) akin to grazing-incidence light fields. The propagation characteristics of THz SPPs along the metal-air interface can be described by their dispersion relation, i.e., a map between the angular frequency ω and the propagation constant $\beta(\omega)$ of the fundamental mode. When periodic structures are engraved on the metal, surface waves resembling the behavior of SPPs, so-called spoof SPPs [1], can be still sustained, and their dispersion relation can be tailored by varying the geometry of the periodic structures. For a *metal-insulator-metal* plasmonic waveguide structure [2] with periodic grooves engraved on both metallic surfaces (counter-facing towards each other), so-called a *spoof-insulator-spoof* (SIS) waveguide structure, the analytical expression of its dispersion relation is given by [3]:

$$\beta = k_0\sqrt{1+\frac{2w}{pgk_0}\tan\left(k_0 d\right)} \ , \tag{S1}$$

where $k_0 = \omega/c$ denotes the propagation constant of plane waves in free space, c being the speed of light. The width w, depth d, and period p describe the geometry of the grooves, where g is the size of the air gap that separates the two flat metallic surfaces. When the duty cycle w/p is equal to 0 (or the depth d is 0), this condition indicates that there are no grooves etched on the metal, and thus, the dispersion relation has an exact linear dependence on the frequency (i.e., no dispersion). When the geometry of the grooves is in the subwavelength scale, the effective medium theory can be applied, thus the effective refractive index n_{eff} is expressed as [3]:

$$n_{eff} \equiv \frac{\beta}{k_0} = \sqrt{1 + \frac{2w}{pgk_0}\tan\left(k_0 d\right)}. \qquad \text{(S2)}$$

Based on Eqs. S1 and S2, it is clear that the propagation characteristics of the spoof SPPs can be easily tuned by engineering the depth of the grooves d, the duty cycle w/p, as well as the gap size g between the two metal surfaces.

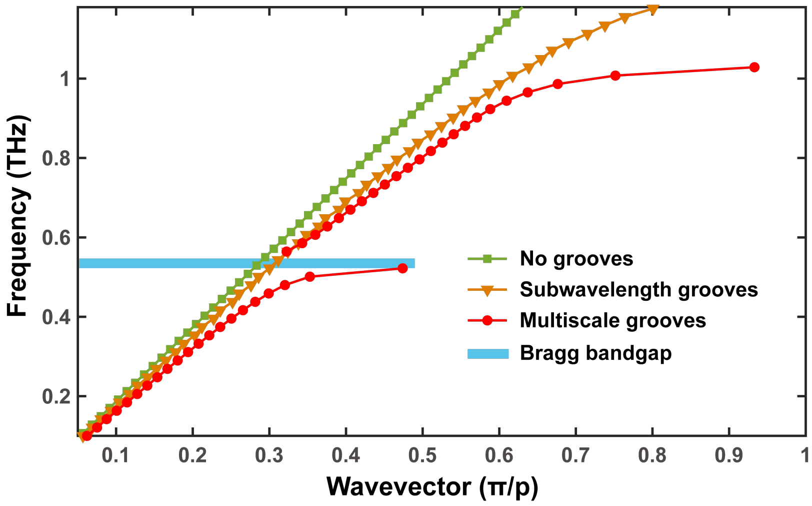

Fig. S2. Simulated dispersion relations for the plain TWWG (no grooves) and the TWWGs with subwavelength-scale and multiscale grooves.

In principle, the THz guidance in metal-wire waveguides also relies on the propagation of SPPs along the metal-air interface [4]. In our study, we aim to tailor the propagation characteristics of the SPPs confined in-between two metal wires by corrugating the wire surfaces with periodic grooves, as shown in Fig. S1(a). Although such a structure can be also considered as a SIS waveguide, its dispersion relation cannot be directly expressed using Eq. S1. This is because Eq. S1 can only be used to describe the propagation characteristics of the spoof SPPs confined in-between *two plane conductors*. Due to the non-planar surface of the metal wires, the depth of the grooves d and the air gap between the two wires g are *not constant* along the cutting direction (y-direction), in turn making it extremely difficult to derive an analytical expression of the dispersion relation. In order to achieve the most accurate results, finite-difference-time-domain (FDTD)

simulations were performed. The dispersion relation shown in Fig. S2 describes the propagation characteristics of the spoof SPPs for a depth d = 40 µm, a width w = 35 µm, and a period p = 80 µm. The dispersion relation shows a cut-off frequency f_c at ~1.2 THz, demonstrating that the spoof SPPs at such a frequency are stopped (propagation velocity equals 0) and the THz frequencies above f_c cannot be guided within the waveguide. By adjusting the geometry of the grooves to the wavelength scale, the cut-off frequency f_c can be accordingly shifted to the lower frequency range, in turn narrowing the operating bandwidth. Therefore, we could expect that, in principle, it is possible to modify the propagation velocity and the cut-off frequency of the propagating spoof SPPs, by tailoring the geometry of the grooves, where we change the depth d (or duty cycle w/p) while keeping the periodicity; however, with such a procedure, a Bragg resonance cannot be achieved within the operating THz bandwidth.

In order to overcome this issue and introduce a Bragg resonance without influencing the bandwidth, we introduce the concept of multiscale structures into the THz regime. A multiscale structure is achieved by superimposing a ***wavelength-scale*** periodic modulation Λ onto the subwavelength-scale periodic grooves. Such a multiscale structure can be interpreted as the combination of two sub-cells with different propagation constants β_i, where the subscripts $i=1,2$ correspond to each individual sub-cell. Any geometrical difference (depending on d and w/p) between the sub-cells results in different n_{eff}, in turn leading to a periodic modulation at the wavelength scale, as shown in Fig. S1(b). The dispersion relation β_m of the spoof SPPs propagating along such a multiscale structure can be obtained from the Bloch theorem [5]:

$$\cos\left[\beta_m\left(p_1+p_2\right)\right]=\cos\left(\beta_1 p_1\right)\cos\left(\beta_2 p_2\right)-\frac{1}{2}\left(\frac{\beta_1}{\beta_2}+\frac{\beta_2}{\beta_1}\right)\sin\left(\beta_1 p_1\right)\sin\left(\beta_2 p_2\right) \qquad \text{(S3)}$$

In our design shown in Fig. S1(b), we have $p_1=80$ µm, $p_2=60$ µm, and $T=2p_1+2p_2$=280 µm. In particular, for the sub-cell 2, the duty cycle w/p equals 0 (or $d=0$). The solution of Eq. S3 exhibits a bandgap at the irreducible Brillouin zone boundary [3], where $\beta_m=\pi/T$. In this condition, the upper limit of the bandgap edge occurs at $f_{bandgap}$= $\beta_m c/2\pi=c/(2T)$, which also satisfies the Bragg condition. The simulated dispersion relation in Fig. S2 confirms the existence of a Bragg bandgap at 0.53 THz. By simply altering the period of the wavelength-scale modulation Λ, the location of such a Bragg bandgap can be tuned within a bandwidth as large as ~1 THz. Based on Eq. S3, it is clear that the concept of multiscale structures offers more degrees of freedom to tailor the spectral response of the entire structure and, as such, it is considered an effective tool for manipulating the properties of the spoof SPPs that propagate in metal-wire waveguides.

<u>Practical considerations:</u> For sample fabrications, we usually engrave the designed multiscale structures along one of the two wires in the TWWG, in order to avoid the alignment of the grooves on both sides. The misalignment of the grooves can lead to a phase shifts between the THz electric field propagating along the two wires, in turn introducing additional loss on the relatively higher frequency side [6].

Supplementary Note 2: Interface forming with 'A-A' or 'B-B' defects

As shown in Fig. S1(b), our proposed multiscale structure consists of two sub-cells, denoted as A and B. Two multiscale structures, with different topological invariants (Zak phases), can be formed based on either the 'A-B' unit cell or the 'B-A' unit cell. A topological interface is formed when the multiscale structure with 'A-B' unit cell is on the left and the multiscale structure with 'B-A' unit cell on the right, leading to a 'B-B' defect at the center of the interface. This is the case we implement in our manuscript. However, the topological interface can also be formed when the multiscale structure with 'B-A' unit cell is on the left and the multiscale structure with 'A-B' unit cell on the right; in this case, an 'A-A' defect occurs at the center of the interface [7], [8].

As shown in Fig. S3, we investigate the transmission spectra (Fig. S3a) and the THz electric field distributions (Fig. S3b) under both conditions. Despite the fact that the THz electric field distributions exhibit different symmetries around the interface, the transmission spectra with both 'A-A' and 'B-B' defects are exactly the same. It is confirmed that as long as the structures on both sides satisfy mirror symmetry and the Zak phases have a difference of π, the existence of the topological interface is guaranteed. For experiments, we have chosen the 'B-B' defect mainly due to fabrication constrains. Compared to directly engraving four consecutive grooves in the center, it is indeed much easier to skip some regions to form the 'B-B' defect.

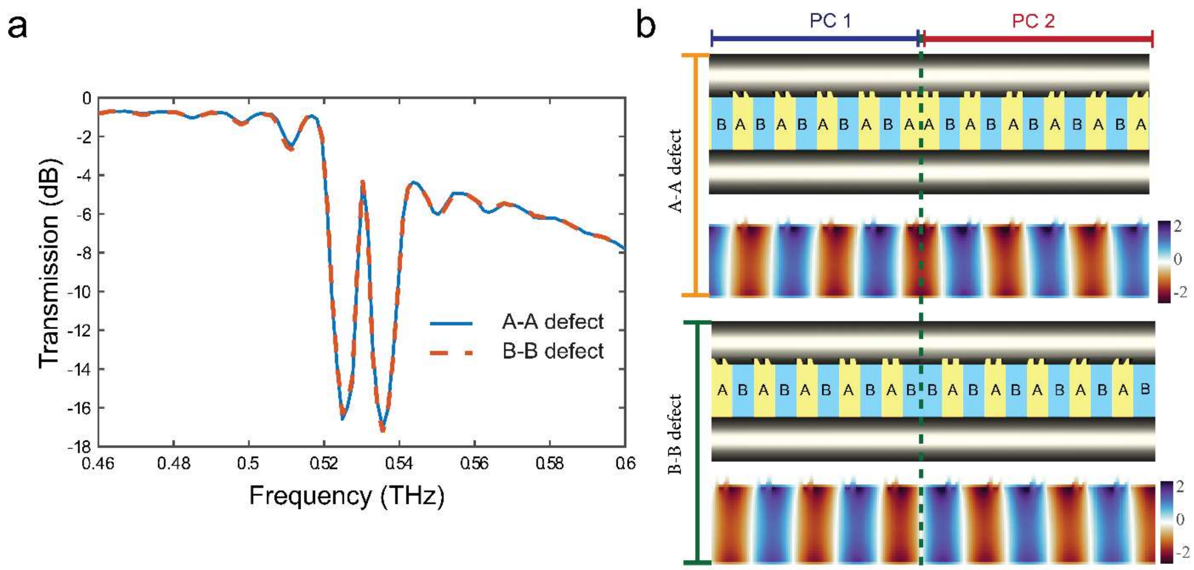

Fig. S3. Comparison between 'A-A' and 'B-B' defect interfaces. (a) Comparison between simulated transmission spectra of the TWWGs with topological interface states based on 'A-A' and 'B-B' defects, respectively. (b) Simulated THz electric field distribution at 0.53 THz of the TWWGs with topological interface states based on 'A-A' and 'B-B' defects, respectively.

References:

[1] J. B. Pendry, “Mimicking Surface Plasmons with Structured Surfaces,” *Science (80-.).*, vol. 305, no. 5685, pp. 847–848, Aug. 2004.

[2] M. A. Kats, D. Woolf, R. Blanchard, N. Yu, and F. Capasso, “Spoof plasmon analogue of metal-insulator-metal waveguides,” *Opt. Express*, vol. 19, no. 16, p. 14860, Aug. 2011.

[3] Q. Zhang, J. J. Xiao, D. Han, F. F. Qin, X. M. Zhang, and Y. Yao, “Microwave band gap and cavity mode in spoof-insulator-spoof waveguide with multiscale structured surface,” *J. Phys. D. Appl. Phys.*, vol. 48, no. 20, 2015.

[4] S. A. Maier, S. R. Andrews, L. Martín-Moreno, and F. J. García-Vidal, “Terahertz Surface Plasmon-Polariton Propagation and Focusing on Periodically Corrugated Metal Wires,” *Phys. Rev. Lett.*, vol. 97, no. 17, p. 176805, Oct. 2006.

[5] S. V. Zhukovsky, A. A. Orlov, V. E. Babicheva, A. V. Lavrinenko, and J. E. Sipe, “Photonic-band-gap engineering for volume plasmon polaritons in multiscale multilayer hyperbolic metamaterials,” *Phys. Rev. A*, vol. 90, no. 1, p. 013801, Jul. 2014.

[6] J. Dong *et al.*, “Versatile metal-wire waveguides for broadband terahertz signal processing and multiplexing,” *Nat. Commun.*, vol. 13, no. 1, p. 741, Feb. 2022.

[7] A. Blanco-Redondo *et al.*, “Topological Optical Waveguiding in Silicon and the Transition between Topological and Trivial Defect States,” *Phys. Rev. Lett.*, vol. 116, no. 16, p. 163901, Apr. 2016.

[8] J. Wang *et al.*, “Topologically tuned terahertz confinement in a nonlinear photonic chip,” *Light Sci. Appl.*, vol. 11, no. 1, p. 152, Dec. 2022.
